# Sex-Differential Herbivory in Androdioecious *Mercurialis annua*

**DOI:** 10.1371/journal.pone.0022083

**Published:** 2011-07-13

**Authors:** Julia Sánchez Vilas, John R. Pannell

**Affiliations:** Department of Plant Sciences, University of Oxford, Oxford, United Kingdom; Umea University, Sweden

## Abstract

Males of plants with separate sexes are often more prone to attack by herbivores than females. A common explanation for this pattern is that individuals with a greater male function suffer more from herbivory because they grow more quickly, drawing more heavily on resources for growth that might otherwise be allocated to defence. Here, we test this ‘faster-sex’ hypothesis in a species in which males in fact grow more slowly than hermaphrodites, the wind-pollinated annual herb *Mercurialis annua*. We expected greater herbivory in the faster-growing hermaphrodites. In contrast, we found that males, the slower sex, were significantly more heavily eaten by snails than hermaphrodites. Our results thus reject the faster-sex hypothesis and point to the importance of a trade-off between defence and reproduction rather than growth.

## Introduction

Plants with separate sexes are often sexually dimorphic, with males and females differing in secondary sexual traits such as inflorescence architecture, shoot morphology, plant life history, water relations, plant size, and susceptibility to herbivory [1]. A particularly common pattern is for males to be more prone to herbivory than females [2]. For instance, of eleven species studied for differential levels of damage to flowers and inflorescences, all showed greater levels of damage in males [3]. Similar patterns have been found for leaves, particularly for herbivory by invertebrates [4], [2]. The tendency for individuals with a male function to be more prone to herbivory extends beyond species with purely separate sexes, and appears to apply just as much to gynodioecious species, in which hermaphrodites are more susceptible than females [5]. In populations where hermaphrodites vary quantitatively in their sex allocation, those with greater male allocation tend to suffer more from herbivory [6].

Several hypotheses have been advanced to explain male-biased herbivory in plants. One idea is that males are less well defended than females [7]; another is that they offer better quality food to herbivores and are thus either simply more attractive, or confer greater survivorship on their herbivores [8]; yet another is that they are more apparent. There is as yet only limited direct evidence for these proximate explanations (but see [9], [10], [11], [12]). Nevertheless, each can essentially be conceptualised in terms of life-history trade-offs between growth, reproduction and defence. In particular, if males allocate more to growth than females, this might be traded off against defence, rendering males more vulnerable to herbivore attack. Similarly, slower growing individuals are expected to allocate more to defence and protection than those that are faster growing; we might label this idea the faster-sex hypothesis [13], [7]. Given that males are very often larger than females, and assuming that males do not germinate earlier than females, the faster-sex hypothesis to explain greater herbivory in males, and hypotheses invoking differential trade-offs between the sexes in general, have sound empirical support – at least for species with larger males.

Although males tend to be the larger sex for woody perennials, females are often larger among herbaceous species [14], [15]. Under the faster-sex hypothesis, dioecious species with female-biased size dimorphism ought to show a reversal in the pattern of differential susceptibility to herbivory, i.e., females should be more prone to attack than males. To our knowledge, this hypothesis has never been explicitly tested. Here, we test this idea by subjecting males and hermaphrodites of an androdioecious population of the European plant *Mercurialis annua* (Euphorbiaceae) to herbivory by snails. *M. annua* is a wind-pollinated annual-herb that occupies disturbed habitats throughout central and western Europe and around the Mediterranean Basin [16], [17]. In the Iberian Peninsula and Morocco, populations are often androdioecious, i.e., with separate male and hermaphrodite individuals [18] and male frequencies range between zero and about 0.4 [16], [19], [20]. Androdioecious populations of *M. annua* are known to be sexually dimorphic in size, with hermaphrodites rather than males being the larger sex, and are often subject to moderate levels of herbivory by snails (personal observation). The faster-sex hypothesis thus predicts that hermaphrodites of *M. annua* should be more prone to damage by herbivores than the slower-growing males. Our study also represents the first analysis of differential herbivory between the sexes for an androdioecious species.

## Materials and Methods

### Experiment

Seeds for the experiment were collected from an androdioecious population near Fes (Morocco), in which the male frequency was approximately 0.45 [21]. Seedlings were first reared in germination trays until they began to flower, approximately four weeks after sowing. Pairs of plants, one male and one hermaphrodite, were then transplanted into each of 80 20 cm diameter pots filled with peat-based soil (Astro Universal, Goundrey's Oxford, UK). All pots were enclosed in perforated bags to exclude (or retain) herbivores. When plants were five weeks old, half the pots were allocated to a herbivory treatment, and the remainder served as controls. The herbivory treatment involved placing two adult brown garden snails, *Helix aspersa* (Helicidae) onto the soil midway between the two plants in the appropriate pots; the snails were from gardens in Oxford and were starved for five days prior to the experiment. Although *M. annua* in the Iberian Peninsula and Morocco is commonly attacked by snails of the genus *Cepaea*, *H. aspersa* is a generalist herbivore that is occasionally found on dioecious *M. annua* in Britain. *Mercurialis annua*, as well as its close relative *Mercurialis perennis*, contains large amounts of the alkaloid hermidin [22], [23]. Although the defensive role of this particular alkaloid is not known, alkaloids are common defensive compounds against generalist herbivores [24]. In addition, the production of alkaloids is metabolically expensive and correlates negatively with measures of plant growth [25]. Our experiment thus addresses the response to herbivore damage in *M. annua* by a generalist herbivore.

We measured the height of each plant and the size of the snails immediately before the experiment. After two weeks, we recorded the height of each plant again and counted the number of damaged and undamaged leaves. For all damaged leaves, we estimated the proportion of leaf area damaged by herbivory using graph paper. We then calculated the average percentage of leaf damage to damaged leaves (total proportion of leaf area damaged/total number of damaged leaves ×100), and the percentage of plant damaged (total proportion of leaf area damaged/total number of leaves ×100). Finally, we measured the oven-dried above-ground biomass of each plant.

### Analysis

We performed linear mixed-effect models to test for differences between males and hermaphrodites in their response to the herbivory treatment. Pot was treated as random effect in the analysis. Initial height was included as covariate, but removed in the analysis of the number of damaged leaves and average percentage of leaf damage to damaged leaves, where it was far from statistical significance (*P* = 0.254 and *P* = 0.304, respectively). In order to account for variation in damage due to snail size, we initially also included the initial size of the snails as a covariate. However, snail size was always non-significant (*P*>0.20) and was not included in the final model. Total dry mass and number of damaged leaves were square-root transformed, and average percentage of leaf damage to damaged leaves, percentage of plant damaged and total number of leaves were all log_10_-transformed to achieve Normality of standardized residuals and homogeneity of variance. Test of significance were carried out using *F*-tests, based on marginal sums of squares. As noted above, prior to testing the main factors, non-significant interactions were removed from the model (*P*>0.200). All analyses were performed in R v. 2.8.1 (R Development Core Team 2008) using the lme function from the package nlme [26].

## Results

**Figure 1:**
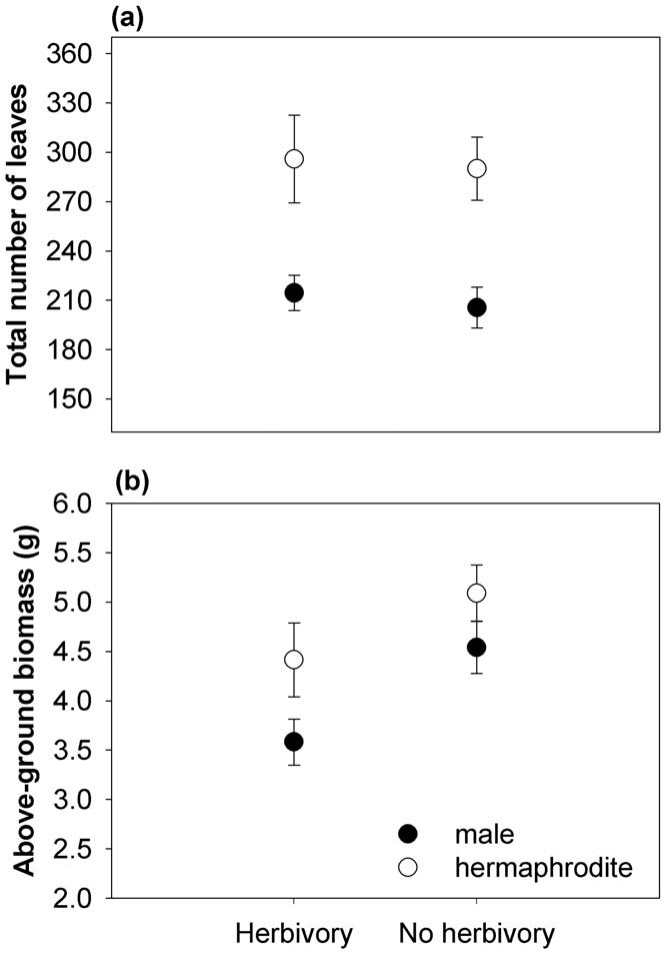
Total number of leaves (a) and above-ground biomass (b) of male and hermaphrodite plants of *M. annua* growing with and without herbivores. Values are means ± SE (N = 34).

**Figure 2:**
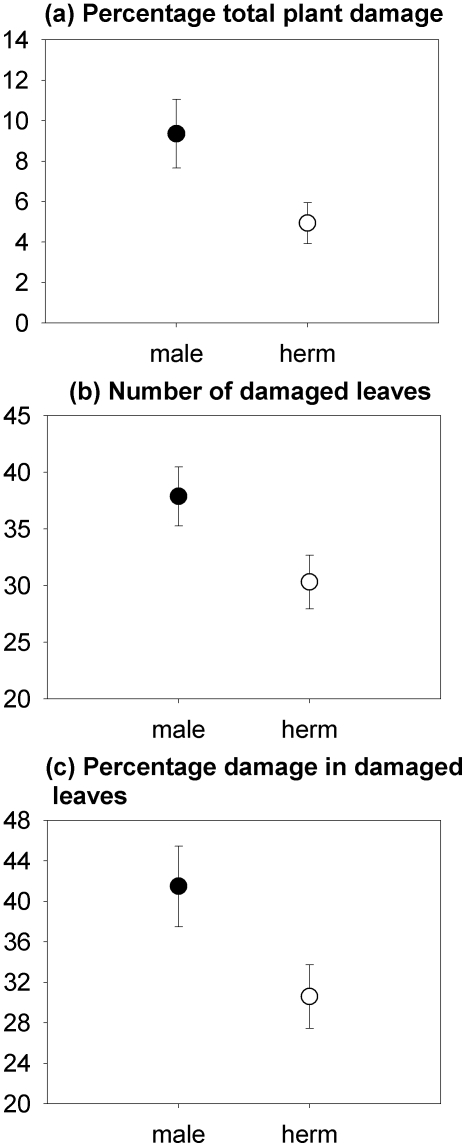
Percentage of total plant damage (a), number of damaged leaves by plant (b) and percentage of leaf damage to damaged leaves (c) in males (“male”) and hermaphrodites (“herm”) of *M. annua* after exposure to snail's herbivory. Values are means ± SE (N = 39).

Hermaphrodites had more leaves and greater total above-ground biomass than males, regardless of the herbivory treatment (Table 1, Figure 1). Herbivory reduced the above-ground biomass of both males and hermaphrodites similarly (Table 1, Figure 1b), but males experienced greater proportional plant damage by herbivores than hermaphrodites (*F*_1,37_ = 18.2, *P*<0.001; Figure 2a), both in terms of the number of damaged leaves (*F*_1,38_ = 6.35, *P* = 0.016; Figure 2b), as well as the average percentage of leaf damage to damaged leaves (*F*_1,38_ = 8.19, *P* = 0.007; Figure 2c).

**Table 1:** Results of the mixed effect models for the total dry mass and the number of leaves.

		Total dry mass		Number of leaves	
Source of variation	df	F	P	F	P
Height	1,76	84.1	<0.001	14.1	<0.001
Herbivory	1,76	9.22	0.003	0.114	0.737
Sex	1,76	22.2	<0.001	22.6	<0.001
Herbivory×Sex	1,75	0.223	0.638	0.582	0.448

Pot was included as random variable in the analysis and the other variables were treated as fixed. P-values for main factors were obtained after removing non significant interaction term from the model.

## Discussion

We found that males were eaten by herbivores more than were hermaphrodites. We also found that males were smaller than hermaphrodites, confirming the previous finding of sexual size dimorphism in *M. annua*. Our results therefore reject the faster-sex hypothesis for sex-differential herbivory: in *M. annua*, it is the slower-growing sex, *i.e.*, males, that is more strongly targeted by herbivores than the faster sex. Instead, our results are consistent with the majority of previous studies that have demonstrated herbivore attack biased towards males in dioecious species and towards pollen-bearing morphs (i.e. hermaphrodites) in gynodioecious species [5]. In other words, our results are consistent with the view that individuals with a primarily male function are more prone to herbivory, whether or not they are the faster-growing sex. The faster-sex hypothesis is based on the idea of trade-offs between growth and defence: individuals that allocate resources to rapid growth are less well defended against herbivores. Given that a growth-defence trade-off evidently does not explain male-biased herbivory in *M. annua*, to what can we attribute the pattern?

The most likely explanation for the observed male-biased herbivory in *M. annua* is a trade-off between *reproduction* and defence. Previous work with dioecious *M. annua*, which has a very similar pattern of sex allocation to that displayed by androdioecious populations of the species, explained the smaller size of males in terms of a trade-off between allocation to growth versus reproduction, both because males begin to flower earlier than hermaphrodites and because of ongoing (indeterminate) allocation to flowering, particularly in terms of nitrogen [14], [27]. Because pollen is rich in nitrogen, selection to enhance their pollen production causes them to allocate more nitrogen to reproduction than females or hermaphrodites [14]. Males of *M. annua* have higher root∶shoot ratios than females or hermaphrodites, perhaps in response to selection to enhance the uptake of nutrients required for reproduction [14], [27]. Nevertheless, foliar nitrogen content in *M. annua* tends to be lower in males than females [27]. It is possible, therefore, that males compromise their production of (N-based) secondary metabolites, which might be important in defence against herbivores, in order to invest in reproduction. It is also possible that higher damage in males is the outcome of increased consumption by the herbivores to compensate for the low nutritional quality of the leaf tissues [2]. However, since snails could freely choose between a male or an hermaphrodite plant, this explanation seems unlikely.

Selection to enhance plant reproductive success, whether through male or female functions, must always operate under trade-off constraints that are more than two-dimensional. It has long been accepted that growth and reproduction trade off against one another, and the faster-sex hypothesis tested here posits a trade-off between growth and defence. Our study suggests that, in *M. annua*, the greater damage sustained by males to herbivore attack is likely the result of a reproduction-defence trade-off, i.e., that reproduction compromises both growth and defence simultaneously.

The current study indicates that *M. annua* shows sexual size dimorphism that is not affected by herbivory. This result may indicate that males are capable of growth compensation in response to herbivory, but also that males may have greater proportion of biomass allocated to structures that enhance pollen dispersal [20], [28]. In fact, males of *M. annua* produce their flowers on long peduncles that are held above the plant, and hermaphrodites produce them in the axils of leaves. Either way, greater damage to leaves is likely to cause reduced allocation to reproduction [5]. It is not yet known to what extent allocation to defence in *M. annua* is induced, i.e., whether herbivory causes the up-regulation of defence-compound production, as is known for a wide range of other species [29]. If so, the cost of defence itself, e.g., in terms of growth, but particularly in terms of reproduction, should also depend on the presence or absence of herbivores.

Sex-biased herbivore preference or tolerance may have important consequences for sexual-system evolution through its effects on the distribution of plant sex allocation and realised gender. Herbivory is known to affect the sex expression of hermaphrodites, shifting their allocation towards female or male function, depending on the species and on whether damage is to leaves or to reproductive organs [5]. Such shifts in sex allocation occur in hermaphrodite plants of monomorphic populations, but also in hermaphrodites of gynodioecious species and androdioecious species, where females or males, respectively, coexist with hermaphrodites. In gynodioecious *Fragaria virginiana*, for example, herbivory affects mainly to flowers of hermaphrodites, causing the loss of a greater proportion of male than female functioning flowers that shifts hermaphrodite sex expression towards greater femaleness. This favours selection against females constraining the transition from gynodioecy to dioecy [30]. In sub-androdioecious *Sagittaria lancifolia*, herbivory of mainly the staminate flowers of both males and hermaphrodites shifts male/hermaphrodite relative siring success in favour of males, contributing to their maintenance [31]. In contrast with this latter example, our results suggest that male-biased herbivory in androdioecious *M. annua* is likely to *reduce* male/hermaphrodite relative siring success, giving rise to lower male frequencies in the progeny of populations subject to high levels of herbivory. A direct affect of herbivory on the sex ratios in natural plant populations now awaits confirmation.
